# Hymenoptera Allergens: From Venom to “Venome”

**DOI:** 10.3389/fimmu.2014.00077

**Published:** 2014-02-28

**Authors:** Edzard Spillner, Simon Blank, Thilo Jakob

**Affiliations:** ^1^Immunological Engineering, Department of Engineering, Aarhus University, Aarhus, Denmark; ^2^Center of Allergy and Environment (ZAUM), Helmholtz Center Munich, Technical University, Munich, Germany; ^3^Allergy Research Group, Department of Dermatology, Medical Center, University of Freiburg, Freiburg, Germany

**Keywords:** allergen components, allergy, cross-reactivity, insect venom, recombinant allergens, sensitization

## Abstract

In Western Europe, Hymenoptera venom allergy (HVA) primarily relates to venoms of the honeybee and the common yellow jacket. In contrast to other allergen sources, only a few major components of Hymenoptera venoms had been characterized until recently. Improved expression systems and proteomic detection strategies have allowed the identification and characterization of a wide range of additional allergens. The field of HVA research has moved rapidly from focusing on venom extract and single major allergens to a molecular understanding of the entire “venome” as a system of unique and characteristic components. An increasing number of such components has been identified, characterized regarding function, and assessed for allergenic potential. Moreover, advanced expression strategies for recombinant production of venom allergens allow selective modification of molecules and provide insight into different types of immunoglobulin E reactivities and sensitization patterns. The obtained information contributes to an increased diagnostic precision in HVA and may serve for monitoring, re-evaluation, and improvement of current therapeutic strategies.

## Hymenoptera Venom Allergy

Hymenoptera venom allergy (HVA) is defined as systemic allergic or anaphylactic reactions that occur in response to stings of insects of the Hymenoptera order. In central and western Europe, this involves most commonly stings by yellow jackets and honey bees, and less frequently stings by hornets or bumble bees. In southern parts of Europe, paper wasps (Polistinae) also play a relevant role. The prevalence of systemic allergic reactions to Hymenoptera stings ranges from 0.3 to 3.4% in the general population. The lowest occurrence is reported in children and the highest in beekeepers (1). Data extrapolated from hospital admissions and emergency department visits (2–5) as well as from a national register for anaphylaxis (6) suggest that HVA may account for up to one third of all anaphylactic reactions. The diagnosis of HVA is routinely based on the clinical history and detection of immunoglobulin E (IgE)-mediated sensitization by skin testing and/or by *in vitro* detection of venom-specific IgE. In addition, cellular test such as basophil activation test or histamine release test are used to identify the sensitizing venom in cases in which the routine testing is not conclusive. Once the diagnosis is confirmed, immunotherapy with the culprit venom offers a high degree of protection from future anaphylactic sting reactions ranging from 80 to 84% in bee venom allergy and 90–95% in yellow jacket venom (YJV) allergy (1).

**Figure 1:**
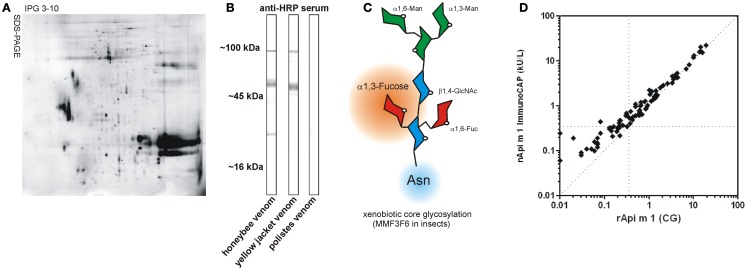
**Venom components and CCD**. **(A)** Representative 2D-gel of the honeybee venom stained with Coomassie Brilliant Blue G-250 demonstrating the complexity of the venome (kindly provided by Dr. Nico Peiren, Laboratory of Zoophysiology, Ghent University, Belgium). **(B)** CCD sIgE-reactivity of rabbit anti-HRP serum with different Hymenoptera venoms in immunoblotting. Note the lack of CCD-reactivity in *Polistes* venom. **(C)** Schematic representation of xenobiotic core glycosylation as found in insects. This carries an additional α 1,3-linked fucose residue compared to plants having an additional β 1,2-linked xylose residue. (GlcNAc, *N*-acetylglucosamine; Man, mannose; Fuc, fucose). **(D)** Comparison of IgE antibody levels to glycosylated rApi m 1 (CG) and native purified Api m 1 in CCD negative HBV allergic patients (*n* = 89). Hatched horizontal and vertical lines indicate the 0.35 kUA/L cut-off and the hatched diagonal line represents a 1:1 ratio.

For diagnostic as well as for therapeutic purposes, whole venom preparations are generally used (Figure 1A). All diagnostic systems that use whole venom preparations are potentially hampered by IgE cross-reactivity that does not allow a precise distinction between true double sensitization and cross-reactivity between different venoms. This cross-reactivity may be based on IgE-reactivity to homologous single venom allergens present in venom of different families or on IgE-reactivity to cross-reactive carbohydrate determinants (CCD) (7).

Until recently, the limited information on single venom allergens and their unavailability for diagnostic and therapeutic purposes rendered HVA an outmoded field, particularly when compared to the progress made in the molecular understanding of other forms of allergies.

Recent advances in expression systems and proteomic detection strategies have allowed the identification and characterization of a wide range of additional Hymenoptera allergens and have moved the field rapidly from focusing on whole venoms and single major allergens to a molecular understanding of the entire “venome” as a system of unique and characteristic components. Here, we review the current information on venom allergens in different Hymenoptera species, their use for reliable diagnostic detection of HVA as well as their potential role in therapeutic intervention.

## Hymenoptera Venom Allergens

Understanding hypersensitivity reactions to venom allergens is often hampered by complexity of the source material (Figure 1A). This is best exemplified by the venom allergen components of the honeybee (*Apis mellifera*) and the common yellow jacket (*Vespula vulgaris*), as they are known to date (Table 1).

**Table 1:** **Overview about the presently known Hymenoptera venom allergens**.

Allergen	Name/function	MW (kDa)	% DW	Potential N-glycosylation	Eukaryotic expression
**BEES (*Apis mellifera, A. cerana, A. dorsata*)**					
Api m 1, Api c 1, Api d 1	Phospholipase A2	17	12	1	+
Api m 2	Hyaluronidase	45	2	3	+
Api m 3	Acid phosphatase	49	1–2	2	+
Api m 4	Melittin	3	50	0	−
Api m 5	Allergen C/DPP IV	100	<1	6	+
Api m 6	Protease inhibitor	8	1–2	0	+
Api m 7	Protease	39	?	3	+
Api m 8	Carboxylesterase	70	?	4	+
Api m 9	Carboxypeptidase	60	?	4	+
Api m 10	CRP/icarapin	55	<1	2	+
Api m 11.0101	MRJP 8	65	?	6	+
Api m 11.0201	MRJP 9	60	?	3	+
Api m 12	Vitellogenin	200	?	1	+
**BUMBLEBEE (*Bombus pennsylvanicus, B. terrestris*)**					
Bom p 1, Bom t 1	Phospholipase A2	16		1	−
Bom p 4, Bom t 4	Protease	27		0, 1	−
**YELLOW JACKETS (*Vespula vulgaris, V. flavopilosa, V. germanica, V. maculifrons, V. pensylvanica, V. squamosa, V. vidua*)**					
Ves v 1, Ves m 1, Ves s 1	Phospholipase A1	35	6–14	0, 0, 2	+
Ves v 2.0101, Ves m 2	Hyaluronidase	45	1–3	4	+
Ves v 2.0201	Hyaluronidase^a^	45	?	2	+
Ves v 3	DPP IV	100	?	6	+
Ves v 5, Ves f 5, Ves g 5, Ves m 5, Ves p 5, Ves s 5, Ves vi 5	Antigen 5	25	5–10	0	+
Ves v 6	Vitellogenin	200	?	4	+
**WHITE-FACED HORNET, YELLOW HORNET (*Dolichovespula maculate, D. arenaria*)**					
Dol m 1	Phospholipase A1	34		2	−
Dol m 2	Hyaluronidase	42		2	−
Dol m 5, Dol a 5	Antigen 5	23		0	+
**HORNETS (*Vespa crabro, V. magnifica, V. mandarinia*)**					
Vesp c 1, Vesp m 1	Phospholipase A1	34		0	−
Vesp ma 2	Hyaluronidase	35		4	
Vesp c 5, Vesp ma 5, Vesp m 5	Antigen 5	23		0	−
**EUROPEAN PAPER WASPS (*Polistes dominula, P. gallicus*)**					
Pol d 1, Pol g 1	Phospholipase A1	34		1	−
Pol d 4	Protease	33		6	−
Pol d 5, Pol g 5	Antigen 5	23		0	−
**AMERICAN PAPER WASPS (*Polistes annularis, P. exclamans, P. fuscatus, P. metricus*)**					
Pol a 1, Pol e 1	Phospholipase A1	34		0	−
Pol a 2	Hyaluronidase	38		2	−
Pol e 4	Protease	?			
Pol a 5, Pol e 5, Pol f 5, Pol m 5	Antigen 5	23		0	+
**FIRE ANTS (*Solenopsis invicta, S. geminata, S. richteri, S. saevissima*)**					
Sol i 1	Phospholipase A1	35	<1	3	−
Sol i 2, Sol g 2, Sol r 2, Sol s 2		14		0	+
Sol i 3, Sol g 3, Sol r 3, Sol s 3	Antigen 5	26		2	+
Sol i 4, Sol g 4		12		0	−

*CRP, carbohydrate-rich protein; DPP IV, dipeptidyl peptidase IV; DW, dry weight; MRJP, major royal jelly protein*.

*^a^ inactive isoform*.

The most prominent honeybee venom (HBV) allergens include phospholipase A2, hyaluronidase, and the basic 26 amino acid peptide melittin (8), all of which constitute higher abundance proteins with estimated amounts of 12, 2, and 50% of the venom dry weight (DW), respectively (9). Classical YJV allergens are phospholipase A1, hyaluronidase, and antigen 5 (10), the function of which remains unknown. These two sets of proteins are found with modifications throughout most Hymenoptera species and by far most identified allergens correspond to these protein classes.

In recent years, however significant progress has been made in identification of novel molecules of lower abundance. For some the allergic potential had already been described, such as the acid phosphatase of HBV (Api m 3), however the gene was identified and recombinantly expressed only recently (8, 11). Moreover, with the identification of the 100 kDa allergen C of HBV and its YJV homolog as dipeptidyl peptidase s IV, a novel class of Hymenoptera venom enzymes could be described (12, 13). In YJV in addition to the classical hyaluronidase (Ves v 2.0101), an inactive isoform (Ves v 2.0201), was identified, which seems to be the dominating isoform in the venom (14). Furthermore, it was demonstrated that Api m 10 represents a novel major allergen of HBV with potentially high impact for diagnostic and therapeutic applications (15, 16). Other IgE-reactive proteins of HBV include a putative protease inhibitor (17, 18), a protease (19), an esterase, and a peptidase whose relevance is currently investigated. The newest allergens are the two major royal jelly proteins (MRJP) 8 and 9 (two isoforms of Api m 11) in HBV (20) as well as novel pan-allergens, the vitellogenins Api m 12, and Ves v 6 (21).

In addition to these components with documented allergenic nature, recently some other components such as a C1q-like protein (22), a platelet derived growth factor (PDGF)/vascular endothelial growth factor (VEGF)-like protein (23), and hexamerin (24) were identified, the allergenic nature of which still has to be evaluated.

Transcriptomics very recently suggested the presence of an antigen 5 like protein in the venom of winter bees (25). Even the season (and most likely the climate and geographic region) seem to have profound impact on the venome. Proteomics revealed the presence of the antimicrobial peptide apidaecin (25) further demonstrating that the complexity of the venome is not restricted to larger proteins. The lower molecular weight (MW) fraction of the venom contains a variety of peptidic components with unique biophysical and clinical characteristics. Their contributions to the sting reaction beyond IgE-reactivity however still need to be addressed.

By increasing application of advanced proteomic, peptidomic, and genomic approaches, the venome and thereby the number of allergens, certainly will significantly increase in the future. The most recent proteomic analysis of honey bee venom (Figure 1A) revealed >100 different components (26). Furthermore, another level of complexity is achieved by the generation of additional isoforms and post-translational modification. All available data however suggest that the apparent plasticity of the venome makes its final definition a never ending story.

As HBV and YJV can be considered prototypic for other Hymenoptera venoms, their composition is reflected in other species including the bumble bee (*Bombus terrestris* and the American *B. pennsylvanicus*), the venom composition of which closely resembles that of the honeybee. Bumble bees gained particular importance for pollination industry workers.

In analogy, venom allergens of diverse other *Vespidae* species such as white-faced hornet (*Dolichovespula maculata*) or the European hornet (*Vespa crabro*) are fairly similar to those of the yellow jacket. Allergy to venom of the phylogenetic more distant paper wasps (Polistinae) is common in North America as well as in Europe, especially in Mediterranean areas. Important *Polistes* species in Europe are *P. dominula* and *P. gallicus*, whereas in Northern America other species such as *P. annularis*, *P. apachus*, *P. exclamans*, *P. fuscatus*, and *P. metricus* are dominant. In the last decades, *P. dominula* has increasingly spread across the North American continent and central and northern parts of Europe. The IgE cross-reactivity between European and American *Polistes* species is described as rather low because they belong to different subgenera. In contrast, cross-reactivity between Polistinae and Vespinae (*Vespula*, *Dolichovespula*, and *Vespa*) venoms and purified venom proteins (27) is frequently observed, especially for *Vespula* and both American and European *Polistes* venom (28).

For all these species, only a limited set of allergens has been identified so far although it is quite likely that all venoms will contain conserved allergens such as hyaluronidases, dipeptidyl peptidases, and vitellogenins that in part contribute to molecular cross-reactivity. Other protein families such as proteases (Api m 7, Pol d 4) show clear molecular differences and it remains open if these proteases will be found in all Hymenoptera venoms.

Moreover, it is widely accepted that IgE cross-reactivity between different insect venoms can be attributed to CCD that are present on a large number of venom allergens (Figure 1B). The only exceptions are apparently venoms of *Polistes* species that seem to lack the alpha 1,3-linked fucose (Fuc) residue that is responsible for IgE-reactivity to CCDs (29).

## Recombinant Allergens for the Diagnosis of HVA

The above mentioned considerations and the entomological diversity of potential culprit insects demand a careful diagnostic algorithm prior to immunotherapeutic intervention. Diagnosis of HVA is based on a history of anaphylactic sting reactions, positive skin test responses, and/or detection of specific IgE to Hymenoptera venom. Positive results in skin and serological tests with conventional venom extracts, however, do not always reflect genuine sensitizations and are frequently caused by clinically irrelevant cross-reactive antibodies. Treatment modalities therefore often include different venoms, resulting in higher costs, increased risk of side effects, and possible *de novo* sensitizations. Molecular approaches are increasingly recognized as elegant way to obtain reliable and detailed diagnostic information. Until recently, only a very limited number of venom allergens such as Api m 1, Api m 4, and Ves v 5 was available either as native or recombinant proteins (30, 31). Their use and the possibility to perform analyses on a molecular level resulted in a clear improvement of diagnostic precision (32, 33). Inherent problems and general considerations however apply for the isolation and production of venom allergens. Even with isolation of high abundance allergens you run the risk of having contaminating residual components in the preparation that may distort the picture at a molecular level.

Applying recombinant technologies, this particular problem does not exist but difficulties rather lie in the establishment of an adequate and efficient production system. The efficiency of the prokaryotic approach is often compromised by the need of extensive folding steps limiting its use to structurally relatively simple and small molecules. In contrast, eukaryotes such as yeast and in particular insect and mammalian cells add oligosaccharides, which are similar but not identical to the glycan of the native allergen and which influence the folding and the immunoreactivity (34). Although early recognized (34), in the last few years expression in insect cells was established as appropriate system for insect venom allergens. The functionality of proteins, the epitope authenticity, and the correct folding of resulting proteins could be demonstrated for a large number of allergens (Table 1) (12, 34, 35).

A common problem of *in vitro* diagnosis of insect venom allergy using venom extracts are patients with double positive test results for HBV and YJV that in our HVA patient cohort from the south west of Germany constitute approximately 45–50% of all cases. This double positivity may reflect true double sensitization to HBV and YJV, or may be based on IgE cross-reactivity.

Immunoglobulin E cross-reactivity may be based on common protein epitopes of homologous allergens of both venoms as described for hyaluronidases, dipeptidyl peptidases, and the new 200 kDa vitellogenin allergens. Alternatively, cross-reactivities can be attributed to IgE antibodies directed against cross-reactive glyco-epitopes of the allergens (7, 36, 37). This is of particular importance, since most HBV and YJV allergens are glycoproteins with one or more of such carbohydrate structures (Table 1).

Causative for the phenomenon of cross-reactivity are IgE antibodies that are directed against an alpha 1,3-linked fuc residue of the *N*-glycan core established by insects and plants (Figure 1C). In plants, additionally a beta 1,2-xylose residue is found at the core glycan to which IgE also can be directed. Such xenobiotic modifications represent highly immunogenic epitopes, which can induce specific immunoglobulin G (IgG) as well as IgE antibodies (38). CCD-specific IgE antibodies have been reported to be responsible for more than 50% of double sensitizations to HBV and YJV (37), complicating the choice of the appropriate therapeutic intervention. The clinical relevance of CCD-reactive IgE antibodies is controversially discussed, but in the case of insect venom allergy appears to be low or non-existing. Accordingly, CCD-carrying glycoproteins can effect mediator release from basophils but do not provoke significant responses in individuals with CCD-specific IgE (39). Nevertheless, anti-CCD IgE represent an undoubted pitfall of *in vitro* allergy diagnostics, since they cause multiple reactivities with any glycosylated plant (food, pollen) or insect venom allergen and thereby interfere with the detection of clinically relevant sensitization to protein epitopes. A prominent example of CCD-based interference with diagnostic precision is the honey bee venom major allergen Api m 1 that carries in its natural form an alpha 1,3-linked fuc on a *N*-glycan core structure and thus is reactive with IgE directed against CCDs. Generation of recombinant forms of Api m 1 that either lack the entire core glycan or only the 1,3 fuc residue, demonstrated a high reliability to detect sensitization to the species-specific protein epitopes as compared to nApi m 1 (Figure 1D) (40).

Molecular diagnosis applying non-glycosylated species-specific allergens such as Api m 1 and Ves v 5 (41–43) and strategies to circumvent the presence of CCDs led to a significant advance in the dissection of true double sensitization versus irrelevant cross-reactivity. The use of Sf9 insect cells from *Spodoptera frugiperda* as expression system results in allergens with functional glycosylation, proper folding, and complete epitope spectrum but not showing any immunologically detectable CCD-reactivity (Figure 1D). This phenomenon is obviously based on the specific absence of alpha 1,3-core fucosylation (35). Resulting CCD-free engineered and correctly folded allergens allow for the first time the assessment of their relevance regardless of their natural glycosylation bypassing complex inhibition analyses. Using CCD-free, correctly folded Ves v 2.0101 and Ves v 2.0201, we were able to clearly demonstrate that hyaluronidases – contrary to previous assumptions – do not play a significant role as major allergens of YJV (35), a fact that was corroborated by findings of others (44, 45). In contrast, even for highly glycosylated proteins such as Api m 5, Api m 10, and Api m 11, a pronounced IgE-reactivity beyond CCDs with clinical relevance was demonstrated (12, 15, 16, 20).

Another problem of *in vitro* diagnosis of HVA using venom extracts are patients with a documented history but negative test results. A possible reason might be that venom extracts represent heterogeneous mixtures in which the components are present in widely varying concentrations and that particular allergens can be lost or degraded during the processing (15). Alternatively, coupling behavior within the assay system or accessibility of individual allergens within the extract may be different from the isolated protein. An excellent example for this kind of discrepancies has been reported quite recently in patients with YJV allergy (46). Among patients with a well-documented history of yellow jacket sting anaphylaxis but negative IgE test results to YJV extract, 84% could be diagnosed by using recombinant Ves v 5 as allergen. Subsequent analysis of a large cohort of YJV allergic patients confirmed that IgE-reactivity to Ves v 5 was under-represented in the whole venom extract. This discrepancy could be solved by spiking the venom extract with rVes v 5, a procedure that was adopted by one of the manufacturers of these diagnostic extracts (Thermo Fisher Scientific). These and many other problems can be bypassed using molecular diagnostics in form of recombinant allergens, which additionally are available in unlimited amounts and thus analytically better accessible.

## Venom Immunotherapy in HVA

Specific immunotherapy with the culprit venom (VIT) offers a high degree of protection from future anaphylactic sting reactions ranging from 80 to 84% in bee venom allergy and 90–95% in YJV allergy (1).

The degree of protection induced by VIT is either extrapolated from patient information on the occurrence and tolerance of field stings or obtained from clinical data in which patients who are receiving maintenance VIT undergo a sting challenge in a controlled clinical setting. A most recent study by Rueff et al. that included more than 1500 patients that had received a sting challenge, observed a protection rate of 84% for bee VIT and 96% for yellow jacket VIT (47). Using a logistic regression model to assess relative risk factors for not being protected, they identified among others VIT with HBV as one of the highest risk factors (as compared to VIT with YJV) with an odds ratio of 5. This difference in honeybee versus yellow jacket VIT has been known for decades and has been suggested to be related to differences in quantities and qualities of venoms that are injected during the sting. The recent progress in the molecular characterization of relevant venoms has demonstrated that in particular in HBV low abundance proteins such as Api m 3, Api m 5, and Api m 10 play an important and until then underestimated role as allergens (11, 12, 15).

Despite the fact that these allergens are present only in low quantities, they must be regarded as major allergens since more that 50% of HBV allergic patients display IgE-reactivity to them (16). Interestingly, two of these allergens, Api m 3 and Api m 10, while present in the crude venom abstract, are absent or under-represented in therapeutic venom preparations (15). When analyzing sensitization profiles in HBV allergic patients, IgE to Api m 3 and/or Api m 10 was detected in up to 68% and in 5% of the patients IgE was directed against Api m 3 and/or Api m 10 only (16). The under-representation of Api m 3 and Api m 10 in therapeutic venom preparations was additionally confirmed indirectly by analyzing allergen sIgG4 in patients under VIT. While VIT induced a robust sIgG4 response to Api m 1, Api m 2, and Api m 4, no or only very little IgG4 induction could be observed to Api m 3 and Api m 10.

Based on these findings, it is tempting to speculate that the relative lack of these two allergens in therapeutic venom preparation may account for the reduced efficacy of VIT in bee venom-allergic patients, a hypothesis that is currently under investigation. Provided that indeed different sensitization profiles to bee venom allergens are associated with an increased risk of not being protected by VIT, one could envision different strategies to improve to efficacy of VIT in these patients. Here improved methods of generating the therapeutic venom preparation may be developed to circumvent the loss of individual allergens. Alternatively, existing venom preparations could be enhanced by spiking with recombinant allergens that are under-represented or lacking. Finally, generation of individual allergen cocktails for patient tailored immunotherapy would be conceivable. The latter two options, however, are highly unlikely, given the relatively small group of patients that would benefit and the high regulatory requirements for registrations of individualized immunotherapy products.

## Conclusion

Advances in the characterization of Hymenoptera venoms offer detailed insights into the molecular basis of toxicology and allergic sensitization potential of individual venom components. Recombinant access and the capability to define the allergen glycosylation allows for advanced strategies for differentiation of genuine double sensitization and cross-reactivity as well as for avoidance of a reactivity bias toward less relevant allergens in extracts. Applying a growing panel of CCD-free species-specific as well as homologous recombinant allergens, molecular diagnosis increasingly allows for establishment of individual sensitization profiles. Such profiles also include the potential to follow the course of therapy, to diagnose therapy-induced *de novo* sensitizations, opportunities to adapt therapeutic intervention, and possibly to develop prognostic markers for therapeutic success.

## Conflict of Interest Statement

Thilo Jakob has received consultancy fees from Thermo Fisher Scientific, Stallergenes, Novartis, and Janssen Cilag; research support from Thermo Fisher Scientific, Dr. Fooke Laboratories, Allergopharma, and Genentech; lecture fees from Thermo Fisher Scientific, ALK-Abello, Bencard, and Essex MSD; and travel support from Thermo Fisher Scientific. Edzard Spillner has received research support from Dr. Fooke Laboratories and Euroimmun and is cofounder of PLS Design GmbH. Simon Blank report no conflicts of interest.

## Abbreviations

BAT, basophil activation test; CCD, cross-reactive carbohydrate determinant; CRP, carbohydrate-rich protein; DPP IV, dipeptidyl peptidase IV; DW, dry weight; ELISA, enzyme-linked immunosorbent assay; Fuc, fucose; GlcNAc, *N*-acetylglucosamine; HRP, horseradish peroxidase; IgE, immunoglobulin E; IgG, immunoglobulin G; Man, mannose; MRJP, major royal jelly protein; MW, molecular weight; PAGE, polyacrylamide gel electrophoresis; PDGF, platelet derived growth factor; sIgE, specific immunoglobulin E; VEGF, vascular endothelial growth factor.
